# Giant cervicothoracic malignant peripheral nerve sheath tumor in neurofibromatosis type I: a case report of integrated management with multimodal guidance

**DOI:** 10.3389/fsurg.2026.1829473

**Published:** 2026-07-23

**Authors:** Xia Yongning, Sang Hongxun, Fan Xiaohai, Li Kuangwen, Duan Wenbo, Cao Lei

**Affiliations:** 1 Department of Orthopaedics, China-Japan Union Hospital of Jilin University, Changchun, Jilin Province, China; 2 Department of Orthopaedics, Shenzhen Hospital of Southern Medical University, Shenzhen, Guangdong Province, China

**Keywords:** *en bloc* resection, malignant peripheral nerve sheath tumor, multidisciplinary approach, neurofibromatosis, vascular reconstruction

## Abstract

**Introduction:**

Neurofibromatosis type 1 (NF1) is a genetic disorder predisposing patients to malignant peripheral nerve sheath tumors (MPNSTs), which represent a leading cause of mortality with poor therapeutic outcomes. Large MPNSTs with NF1 are extremely rare, and only a few clinical reports are published on this disease. We describe a case of giant cervicothoracic MPNST with major vessels encased and present the clinical profiles, radiological findings, operative management, and follow-up data, along with a literature review.

**Case study:**

A 30-year-old male patient complained of an incidentally discovered painful mass in the right neck that had been progressively enlarging, accompanied by right hand weakness and intrinsic muscle atrophy. Contrast-enhanced MRI revealed a giant tumor closely related with the right brachial plexus and intervertebral foramina of C6–T2. The right subclavian artery and internal jugular vein were tightly encased by the tumor. A multidisciplinary approach incorporating advanced 3D imaging, preoperative vertebral artery embolization, and *en bloc* resection with vascular reconstruction achieved successful tumor removal while preserving neurological function. The patient received adjuvant radiotherapy and was followed up on the 6th and 12th months postoperatively. There were no complaints or signs of recurrence.

**Conclusion:**

The successful long-term outcome provides a valuable template for managing similar high-risk cases, though long-term surveillance remains crucial due to the high recurrence potential of MPNSTs. This report contributes to the limited literature on radical surgical management of NF1-associated MPNSTs and emphasizes the critical balance between oncological control and functional preservation in these challenging cases.

## 1 Introduction

Neurofibromatosis type 1 (NF1) is an autosomal dominant disorder caused by mutations in the “NF1” tumor suppressor gene located on chromosome 17q11.2, affecting approximately 1 in 2,500 to 3,500 individuals worldwide (1, 2). This disease is characterized by café-au-lait macules, cutaneous neurofibromas, and skeletal abnormalities, with up to 50% of cases arising *de novo* (3). A critical complication of NF1 is the malignant transformation of plexiform neurofibromas into malignant peripheral nerve sheath tumors (MPNSTs), occurring in 8%–13% of patients and representing the leading cause of mortality in this NF1 population (4, 5). MPNSTs are highly aggressive sarcomas with a 5-year survival rate of 20%–50% and are often resistant to conventional chemical therapies (6, 7). The diagnosis of MPNST remains challenging due to its histological heterogeneity and lack of specific biomarkers and therefore requires a combination of immunohistochemical markers such as S100 and Ki67 for definitive diagnosis (8). Surgical resection with negative margins remains the cornerstone of treatment, though achieving complete resection is often complicated by the close relation between the tumor and major blood vessels and critical nerves (9).

Here we report an apparently rare and clinically challenging scenario: a 30-year-old male with NF1 developed a gigantic cervicothoracic MPNST originating from a pre-existing neurofibroma, as confirmed by immunohistochemistry. This case is notable for its anatomical complexity, including the encasement of subclavian artery and vertebral artery, as well as a symptomatic presentation with progressive neurological deficits (4, 10). Such large, vascularized MPNSTs in critical locations pose significant diagnostic and therapeutic dilemmas, especially in distinguishing benign neurofibromas from malignant transformations and managing surgical risks like catastrophic hemorrhage and irreversible neural impairment (11).

## 2 Case presentation

### 2.1 Patient information

A 30-year-old male complained of a swelling mass in the right neck, accompanied by occasional self-resolving forearm numbness. The mass was initially egg-sized and progressively enlarged to the size of a small ball, accompanied by right hand weakness and intrinsic muscle atrophy. He felt a predominantly decreased endurance for physical activity. Due to the significant impact on the quality of life and inability to work, he came to the clinic for further treatment. He had been diagnosed as Neurofibromatosis Type 1 (NF-1) for over 20 years and underwent posterior spinal deformity correction surgery 13 years ago. The patient's grandfather was diagnosed with NF-1, with typical scattered café-au-lait spots on the skin. His parents and other direct relatives had no history of neurofibromatosis and other genetic disorders.

### 2.2 Clinical findings

Scattered café-au-lait spots were present on the skin, and two ulcerated plexiform neurofibromas were noted in the left scapular region of the back and the left buttock (Figures 1A,B). Physical examination revealed a firm, roughly oval-shaped mass measuring approximately 15 cm × 10 cm in the right neck (Figure 1C). Local tenderness and percussion pain were present, while Tinel's sign was negative. Moderate atrophy of the hand intrinsic muscles and muscle weakness (III/V) were observed.

**Figure 1:**
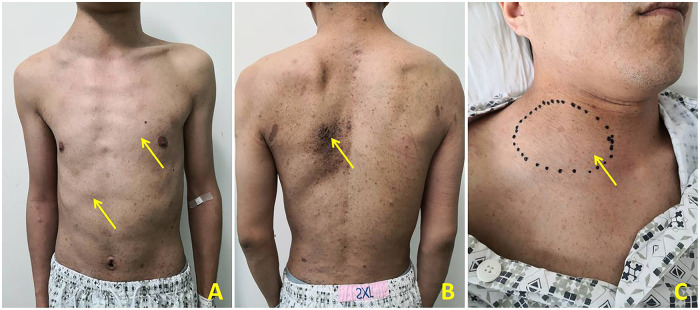
**(A)** Scattered café-au-lait spots on the skin. **(B)** An ulcerated plexiform neurofibroma was noted in the left scapular region. **(C)** A firm, roughly oval-shaped mass measuring approximately 15 cm × 10 cm in the right neck.

### 2.3 Diagnostic assessment

Contrast-enhanced MRI revealed a tumorous lesion in the medial space of the right sternocleidomastoid, measuring approximately 166 mm × 85 mm. The tumor was supplied by the right subclavian artery and right vertebral artery. The right common carotid artery, trachea, and other adjacent structures were compressed to the left. The right subclavian artery and internal jugular vein were tightly encased by the tumor. MRI further demonstrated the tumor's close relationship with the right brachial plexus and intervertebral foramina of C6–T2 (Figures 2A–C). A three-dimensional model was reconstructed on the basis of CT data using Mimic V20.0 (Materialise, Belgium), facilitating visualization of tumor size, morphology, and surgical planning (Figures 2D–F).

**Figure 2:**
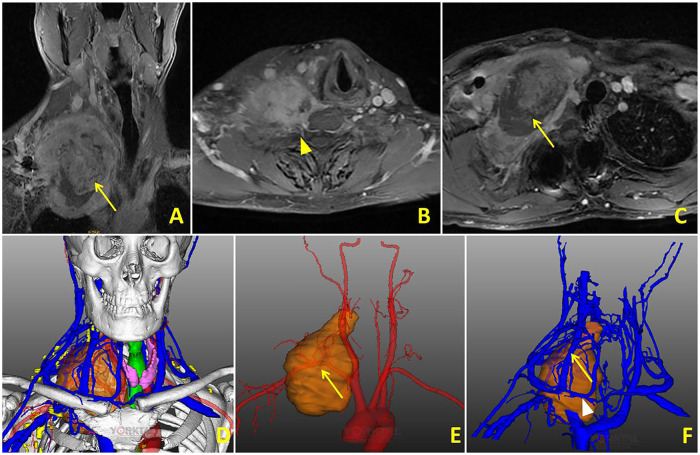
**(A)** Contrast-enhanced MR revealed a giant tumor extending from epiglottis level superiorly to the third intercostal space inferiorly (yellow arrow). **(B)** T2-weighted phase showed the tumor extending into the spinal canal through the intervertebral foramen (yellow triangle). **(C)** Areas of hemorrhage and necrosis within the tumor showed heterogeneous signals (yellow arrow). **(D)** A giant MPNST in the right thoracocervical region with severe adhesion to adjacent structures. **(E)** The clavicular segment of the right subclavian artery (yellow arrow) went through the tumor, with nearly normal physiological function. **(F)** The right internal jugular vein (yellow arrow) was pushed anteriorly, and the right subclavian vein (white triangle) had almost lost its physiological function, with collateral circulation established.

Prior to biopsy, the key clinical and radiological features that distinguished the malignancy from a benign enlarging neurofibroma were: (1) Rapid growth rate within 2 months, (2) Progressive neural function impairment. (3) MRI showed a large, heterogeneous mass with irregular borders and central necrosis-features atypical for benign plexiform neurofibromas, which are typically more homogeneous and well-defined. This suspicion necessitated histological confirmation via core needle biopsy.

Ultrasound-guided needle biopsy pathology indicated a spindle cell malignant peripheral nerve sheath tumor (MPNST). Immunohistochemistry results were as follows: PGP9.5 (+), S-100 (+), SOX10 (−), EMA (−), CK (−), Bcl-2 (+), CD99 (+), Desmin (−), CD34 (−), Dog-1 (−), INI-1 (+), and Ki67 (70%+ in hotspots).

### 2.4 Therapeutic intervention

The patient received right subclavian artery angiography and vertebral artery embolization, followed by surgical tumor resection on the next day. Intraoperatively, the mid-clavicle was osteotomized, and partial sternotomy was performed to expose the tumor. The tumor was measured approximately 140 mm × 90 mm and extended from the epiglottis level superiorly to the third intercostal space inferiorly, with approximately half of its volume intruding into the thoracic cavity. The right subclavian artery, the origin of vertebral artery and phrenic nerve were tightly encased by the tumor and could not be stripped safely. The brachial plexus were compressed posteromedially with severe adhesions to the tumor. The brachial plexus cords were carefully dissected and freed proximally to the root level. After complete dissociation and protection of the peripheral structures (brachial plexus, common carotid artery, vagus nerve, and subclavian vein) (Figures 3A,B), the encased internal jugular vein, right phrenic nerve, clavicular segment of the subclavian artery, and origin of the vertebral artery were ligated and resected *en bloc* with the tumor (Figures 3C–E). A 70 mm × 8 mm Gore-Tex graft (W.L. Gore & Associates, USA) was used for subclavian artery reconstruction, and the remaining internal jugular vein was anastomosed end-to-end directly using 6–0 Prolene suture. The clavicle and sternum were repositioned and fixed with locking plates and nails (Figure 3B). After irrigation and careful hemostasis, a chest tube was placed in the 6th intercostal space at the mid-axillary line. The cervicothoracic incision was closed layer by layer. The whole operation procedure has been recorded and uploaded in Supplementary Video S1. The estimated intraoperative blood loss was approximately 2,500 mL, with 5,550 mL of fluid supplement, 800 mL of plasma, 10 units of red blood cells, and 6 units of cryoprecipitate. Postoperative examination confirmed intact sensation and motor function in all limbs.

**Figure 3:**
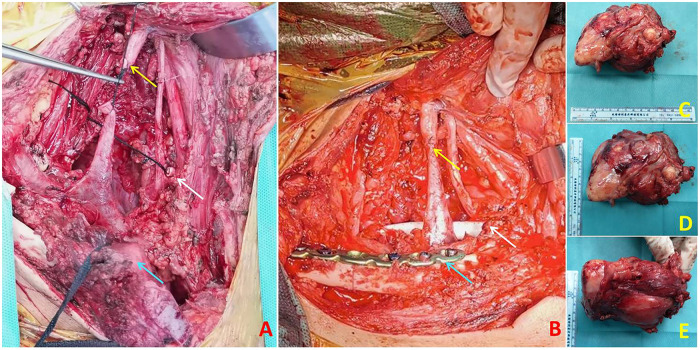
**(A)** During the operation, a clavicle osteotomy was performed (blue arrow) and the tumor was excised together with part of the subclavian artery (white arrow) and internal jugular vein (yellow arrow); **(B)** artificial vascular reconstruction of subclavian artery (white arrow), clavicle reconstruction with titanium internal fixation (blue arrow), and end-to-end anastomosis of internal jugular vein (yellow arrow); **(C)** complete excision of the tumor, measuring approximately 140 mm × 90 mm × 70 mm.

Postoperative pathology confirmed MPNST arising from neurofibroma, with immunohistochemistry results consistent with preoperative findings (Figures 4A–D).

**Figure 4:**
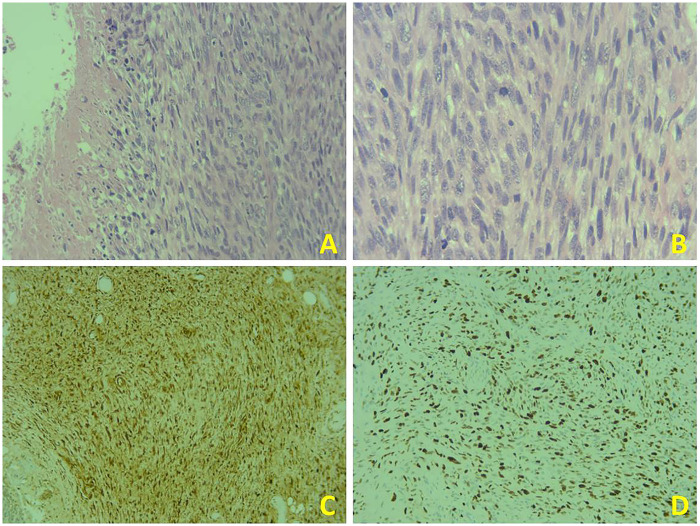
(**A**, 200×) and (**B**, 400×) Hematoxylin and eosin staining shows densely packed spindle-shaped tumor cells arranged in fascicular to storiform patterns, with marked nuclear enlargement and frequent mitotic figures. **(C)** Immunohistochemistry for S-100 demonstrates nuclear staining in tumor cells, indicating the tumors derive from neural sheath. **(D)** Immunohistochemistry for Ki-67 demonstrates high proliferation of the tumor.

### 2.5 Follow-up and outcomes

After surgery, the patient exhibited no sensory deficits in the right upper limb, with motor function unchanged and strong radial artery pulses. Mild Horner's syndrome (ptosis, facial anhidrosis, and enophthalmos) was noted, probably due to intraoperative manipulation of the cervical sympathetic trunk. Postoperative MRI confirmed complete tumor resection, functional artificial subclavicular artery, and intact brachial plexus. 35 sessions of adjuvant radiotherapy was administered after discharge without systemic chemotherapy. At the 6-month and 12-month follow-ups, intrinsic hand muscle strength had improved to grade V, with resolution of facial symptoms and no evidence of tumor recurrence on imaging. The patient's episode of care is listed in Table 1 according to CARE guideline. However, given the limited follow-up period, long-term outcomes remain uncertain. Ongoing follow-up will further evaluate potential recurrence or progress.

**Table 1:** CARE timeline of patient's episode of care.

Time	Event	Key details
November 2024	Symptoms onset	Patient developed a swelling mass in the right neck, accompanied by occasional self-resolving forearm numbness
November 2024	Gradual aggravation	The mass progressively enlarged, accompanied by right hand weakness and intrinsic muscle atrophy
November 11th, 2024	Initial presentation (local hospital)	MRI revealed a large cervicothoracic mass, suspecting a malignant tumor.
December 15th, 2024	Referred to our hospital (tertiary center), preoperative workup	Contrast-enhanced MRI, contrast-enhanced CT and ultrasound-guided needle biopsy; pathology confirmed MPNST
December 24th, 2024	Multidisciplinary team (MDT) discussion	Orthopaedics, thoracic surgery, neurosurgery, vascular surgery, anesthesiology, radiology, oncology jointly formulated the plan.
December 29th, 2024	Vascular intervention	The patient received right subclavian artery angiography and vertebral artery embolization.
December 30th, 2024	Surgical resection	Radical tumor resection achieved (R0). Mild Horner's syndrome was noticed.
February 3^rd^–March 21st, 2025	Adjuvant radiotherapy	The patients received adjuvant radiotherapy (35 sessions) without major complications.
July 2025	6-month follow-up	MRI showed no local recurrence. Neural dysfunction completely resolved.
December 2025	12-month follow-up	No local recurrence or distant metastasis; Patient was in healthy condition and returned to normal life.

### 2.6 Patient's perspective

Before admission, the patient complained of severe persistent painful swelling in the right neck region, accompanied by decreased endurance for physical activity. He felt fatigued after walking approximately 200 m. Due to the significant impact on the quality of life and inability to work, he was admitted to our department for further treatment. After a definitive diagnosis, the patient strongly requested surgery to improve his quality of life. Postoperatively, there was no significant damage to neurovascular function, which gave the patient the confidence and motivation to initiate functional rehabilitation training. At the 12-month follow-up, the patient had fully recovered physical activity capacity and returned to work. He expressed satisfaction with the entire treatment process.

## 3 Discussion

### 3.1 Literature review

Giant cervicothoracic MPNSTs with major blood vessels involved are extremely rare and pose enormous challenges to *en bloc* surgical resection. A systematic literature review was undertaken by searching for articles on PubMed, EBSCO, and ScienceDirect, using the keywords “Malignant Peripheral Nerve Sheath Tumor”. Studies describing MPNST with NF1 have been summarized in Table 2.

**Table 2:** Summary of studies describing MPNST with NF1.

Author	Case NO.	Gender	Age	Location	Clinical presentation	Surgical procedure	Pathological findings	Adjuvant therapy	Follow-up
Yin J, etc. (12)	1	M	70y	Right shoulder, chest wall	Multiple protruding and ulcerated masses	Complete tumor excision	Low-grade MPNST;	Not specified	No recurrence at 9-month follow-up
Bilgic B, etc. (13)	4	3M/1F	31.75y mean	Popliteal, thigh, forearm	Enlarging painful masses with scattered neurofibromas	Complete tumor excision (all cases)	MPNST	Radiotherapy (2 cases) and chemotherapy (1 case)	Two cases died of lung metastases, one died of abdominal MPNST
Petracco G, etc. (14)	1	M	50y	Urinary bladder	Recurrent urinary retention and sepsis	Cistoprostatecomy	MPNST	Not specified	Not specified
Leticia del C, etc. (15)	2	Not specified	Not specified	Case 1: Intraspinal, Case 2: thoracic(T1-T8)	Case1: Pain; Case2: Enlarging painful mass, Thoracic kyphoscoliosis,	Wide resection	MPNST	Not specified	Both died
Terry DG, etc. (16)	1	M	26	Femur (intraosseous)	Painful enlargement of proximal left thigh	Femur resection along with hemiarthroplasty	MPNST	Chemotherapy	Died of multiple metastases 15 months later
Matsunaga k, etc. (17)	1	M	31	Buttock (primary); Cerebral embolism (metastatic)	Acute hemiparesis and unilateral spatial neglect	Mechanical thrombectomy for embolism	MPNST emboli	Not specified	Died of respiratory failure on the 15th day
Chaudhary N, etc. (18)	1	M	10	Paraspinal soft tissue (T3-T11)	Progressive painful swelling over the back	Partial excision	MPNST	Chemoradiotherapy failed; Palliative oral metronomic therapy	remission 20 months post-therapy
Yoo SY, etc. (19)	1	M	Child (Not reported)	GI tract	Abdominal symptoms (not specified) with Moyamoya disease	Tumor resection	MPNST	Not specified	Not specified
Zhang T, etc. (20)	1	F	46	Uterine cervix	Irregular vaginal bleeding	Radical hysterectomy	MPNST	Multi-line chemotherapy	44 months survival
Furuzono N, etc. (21)	1	F	18	Uterine cervix	Abnormal vaginal discharge, prolonged menstruation	Biopsy only (unresectable)	MPNST	Systemic chemotherapy (ineffective)	Died 8 weeks after admission
Zhang X, etc. (22)	1	F	3	Left wrist	Recurrence of soft tissue mass	Two-stage excision	MTT (CDKN2A deletion)	6 cycles of chemotherapy, oral MEK inhibitor	Partial response
Qian L, etc. (23)	1	F	30	Retroperitoneum (with metastases to pelvic wall and muscle)	Back pain and urinary urgency	Palliative resection	MPNST	Not specified	No progression on postoperative day 145
Young SJ (24)	1	M	23	Both liver lobes	Right epigastric pain and jaundice	Autopsy only	MPNST	Not specified	Died 38 days after admission

### 3.2 Epidemiological characteristics

MPNSTs are particularly significant in the context of NF1, as they represent a considerable proportion of soft tissue sarcomas in this population (25). Approximately 50% of MPNST cases are associated with preexisting NF1, highlighting the genetic predisposition that NF1 patients face. Meanwhile, the lifetime risk of developing MPNSTs in NF1 patients is estimated to be as high as 10%, compared to an incidence of only 0.001% in the general population (26). This stark contrast underscores the importance of understanding the epidemiological landscape of MPNSTs, especially in the context of NF1 with plexiform neurofibromas onset. Notably, the risk of malignant transformation from plexiform neurofibromas to MPNSTs is estimated to be between 10%–15%, emphasizing the critical need for vigilant monitoring in this NF1 population (27). The incidence of MPNSTs is multifactorial. Age, tumor size, and genetic predispositions have been proved to be pivotal factors influencing the incidence of MPNSTs (22, 28, 29). In addition, the anatomical location of MPNSTs also influences the incidence and prognosis. MPNSTs occur in the trunk and extremities frequently, with a notable prevalence in areas where large nerve roots converge (26). As malignant sarcomas with complicated predisposing factors, MPNSTs are notorious for their aggressive nature, characterized by high recurrence and metastasis rates. Previous studies indicate that the 5-year survival rate for MPNSTs patients was rarely less than 50% (30).

To conclude, the poor prognosis for patients with MPNSTs is influenced by a myriad of factors, including tumor pathogenesis, pathophysiology, treatment strategies, and patient-specific genetic characteristics.

### 3.3 Pathology and immunohistochemistry

There are no specific MPNST markers for pathology, making diagnosis challenging due to gene mutation heterogeneity and immunohistochemical variability. MPNST, as a nerve-origin solid tumor, is typically white and fleshy and may show myxoid changes. Microscopically, it exhibits various morphologies and distinct subtypes, characterized by elongated, curved nuclei and scant cytoplasm, with common necrosis, mitosis, and hemorrhage (31). Even non-specific, these characteristics aid in differential diagnosis against atypical neurofibroma and nonneurogenic tumors. For example, diffuse cellularity and conspicuous mitoses may ensure the differentiation of MPNST from atypical neurofibroma and nonneurogenic tumors (32).

MPNST lacks distinctive molecular markers and relies on immunohistochemistry for diagnosis. As one of the conventional biomarkers, S-100 is critical for Schwann-associated tumors but is nonspecific due to its 50%–60% positivity rate (33). S-100 negativity may indicate Schwann cell de-differentiation and predict malignancy, so its absence correlates with increased metastasis and mortality risk (34). SOX-10 has similar sensitivity as S-100 in most neural crest-derived tumors, but is poorly sensitive due to its variable expressions in MPNSTs (35). Ki67 levels above 10% may indicate MPNST (36), while CD34-positive cells decrease significantly in high-grade MPNSTs (37, 38). Nestin is a more sensitive marker for MPNST but cannot easily differentiate MPNST from desmoplastic melanoma either (39).

In recent years, several new MPNST markers have been identified in the molecular investigations of MPNST mechanisms. The complete loss of the CDKN2A-encoded cell cycle regulator p16 is a common finding in MPNST. However, the absence of p16 is not malignancy-specific yet (36). p53 protein often accumulates in tumors due to its unique deregulation, and TP53 mutation is linked to high tumor grade in NF1-associated or sporadic MPNST (40), but they can also mislead diagnoses possibly (41). With the function of inhibiting proliferation and promoting apoptosis, p27 staining can be found in 33% of high-grade MPNST (42). However, p53, p16 and p27 lack systematic validation as reliable and specific biomarkers.

The latest MPNST marker is H3K27me3, a downstream target of PRC2 mutation, which plays a key role in the atypical neurofibromatous neoplasm of unknown biological potential (ANNUBP) to MPNST transition. H3K27me3 loss occurs in 30%–90% of MPNST cases, more frequently in sporadic and radiation-associated types than in NF1-associated ones, and may indicate lower survival chances (43, 44). However, H3K27me3 loss does not differentiate MPNST from similar tumors like synovial sarcoma or fibrosarcomatous dermatofibrosarcoma protuberans (43). Recent studies showed that DNA profiling and methylation profiling can be analyzed to distinguish MPNST from benign tumors and facilitate MPNST classification with the help of methylome-based unsupervised clustering (45).

### 3.4 Clinical signs and symptoms

The clinical presentation of these tumors often includes rapidly enlarging masses, which may be accompanied by pain and neurological deficits. Patients typically report symptoms such as localized pain, sensory disturbances, and motor weakness, which can indicate tumor involvement of adjacent neural structures (26). In some cases, the tumor's location and size can lead to more severe complications, such as Horner's syndrome, which arises from sympathetic trunk involvement, or Pancoast syndrome, characterized by shoulder pain and neurological deficits due to brachial plexus compression (46, 47). In addition to the more common presentations, some patients may exhibit atypical symptoms that can complicate diagnosis. For instance, the presence of scoliosis or other musculoskeletal deformities can be associated with NF1 (48, 49) and may mask the underlying presence of a malignancy.

### 3.5 Radiographic signs

MRI is particularly advantageous due to its superior ability to demonstrate neural tissues, allowing for detailed visualization of tumor characteristics (50). MPNSTs often present as fusiform or lobulated masses that follow the course of major nerve trunks, such as the brachial plexus and sciatic nerves. A notable feature is the “tail sign,” where the tumor appears to connect to the nerve at both ends. On T1-weighted images (T1WI), MPNSTs typically show isointense or slightly hypointense signals. Areas of hemorrhage or necrosis within the tumor may show heterogeneous high or low signals, complicating the imaging interpretation (51, 52). Conversely, T2-weighted images (T2WI) usually reveal heterogeneous hyperintensity due to the coexistence of various tumor components, including cellular areas, myxoid stroma, necrotic tissue, and hemorrhage (53). The disappearance of the “target sign,” characterized by a central low signal surrounded by a high signal rim in benign neurofibromas, is a significant indicator of malignancy (54). Studies have demonstrated that MRI can effectively identify indicative features of MPNSTs, such as irregular borders, heterogeneous enhancement, and the presence of denaturation and necrosis (55). Regular imaging surveillance is indispensible in NF1 patients, particularly in those with plexiform neurofibromas, which harbor a significant risk of malignant transformation. Studies indicate that a large proportion of MPNSTs arise from pre-existing nodular lesions, underscoring the importance of vigilant imaging follow-up to identify these changes promptly and guide treatment strategies (50). Emerging imaging technologies, such as positron emission tomography (PET) combined with MRI (PET-MRI) and radiogenomics, have shown promise in enhancing the accuracy of distinguishing between benign and malignant tumors. This combined approach has been suggested to enhance the detection of malignant transformations in PNSTs, thus facilitating earlier intervention and better patient outcomes (50).

### 3.6 Surgical treatments

Surgical intervention remains the cornerstone of treatment for malignant peripheral nerve sheath tumors (MPNSTs), particularly in patients with neurofibromatosis type 1 (NF1). The primary goal of surgery is to achieve complete resection of the tumor with negative margins, as this is crucial for improving prognosis and reducing the risk of recurrence (56, 57). However, the anatomical complexity of tumor locations can pose significant challenges to complete resection. For MPNSTs that infiltrate critical structures, radically resection without compromising vital functions or causing significant morbidity is apparently not feasible (58). Moreover, the surgical approach must be tailored to the individual patient's circumstances, including the size, location, and histological characteristics of the tumor, as well as the patient's overall health status.

Various surgical approaches have been developed to achieve radical tumor resection while maximizing preservation of neurovascular function (59–63). The cervical supraclavicular approach is a traditional surgical technique for tumors located at the cervical root region and the upper thoracic inlet. This approach offers excellent exposure of critical neurovascular structures, such as the brachial plexus and subclavian vessels. However, visualization may be restricted for tumors extending posteriorly beyond the clavicle or into the thoracic cavity, which may necessitate combined approaches (59, 60). The cervicothoracic combined approach is performed via a cervical incision combined with partial sternotomy or posterolateral thoracotomy for dual exposure and *en bloc* removal (64). For tumors that invade the subclavian vessels or brachial plexus, the transmanubrial transclavicular approach is utilized involving resection of the medial clavicle and a portion of the manubrium, thereby facilitating extensive exposure of the brachial plexus and major vessels (65). Given the extraordinary extension (C4–T4 level) of the MPNST in this patient with subclavian vessels and brachial plexus tightly infiltrated, we performed extensive sternotomy and clavicle osteotomy to acquire adequate exposure, thus facilitating meticulous dissection and minimizing the risk of iatrogenic injury.

Even though MPNSTs are well-documented complications of NF1 (4–6), the cervicothoracic location and massive size (140 × 90 × 70 mm, 4,500 g) of this lesion represent an exceptionally rare presentation. Previous literatures typically describe smaller tumors or infiltrate different anatomical regions, such as cervix, uterus, and extremities etc. The rapid growth and neurological deficits observed in this patient align with the classic alarm for malignant transformation NF-1 (66, 67), and the degree of anatomical distortion and critical vascular encasement exceeded most reported cases. Thus, the *en bloc* resection with major vascular reconstruction performed here was undoubtedly necessary. This approach contrasts with recent targeted therapies, which have shown efficacy in reducing tumor burden but are primarily indicated for inoperable plexiform neurofibromas (68, 69).

The concept “en bloc resection” is a gold standard for the MPNST especially in extremities. However, for the malignant lesions that infiltrate vital structure within or adjacent to the mediastinum, no uniform consensus has been achieved. The pathophysiological basis for such surgical challenges lies in the unique tumor microenvironment of NF1-associated lesions. Plexiform neurofibromas and their malignant counterparts are characterized by aberrant vasculature, including arteriovenous malformations and shunts, which significantly elevate hemorrhage risk (70). Furthermore, the underlying molecular pathogenesis of MPNSTs, such as co-deletions in CDKN2A and mutations in TP53, contributes to their aggressive behavior and chemotherapy resistance (71–73). In addition, emerging research highlights intratumoral heterogeneity and the presence of cancer stem-like cells as key drivers of progression and relapse, underscoring the importance of complete surgical eradication when feasible (74). Thus, our adventurous resection strategy is strongly supported by the tumor's malignant pathology and the need for clear surgical margins.

This aggressive yet precise strategy was facilitated by advanced preoperative 3D modeling, which provided an unparalleled understanding of the tumor's relationship with critical neurovascular structures (75). The multidisciplinary team approach, integrating expertise from neurosurgery, vascular surgery, interventional radiology, and oncology, was therefore indispensable for navigating these multifaceted challenges and achieving a favorable oncological and functional outcome. Meanwhile, the decision for preoperative selective vertebral artery embolization, rather than targeting the primary tumor feeders, was an innovation to mitigate the risk of catastrophic intraoperative hemorrhage and sudden cerebral ischemia, which is scarcely reported in prior reports (76). This revolutionary strategy, guided by the tumor's unique vascular anatomy and the presence of functional collateral circulation, exemplifies an unprecedented application of embolization. The subclavicular artery reconstruction and jugular vein anastomosis successfully preserved limb perfusion without sacrificing major vessels. These proactive strategies distinguish this case from previous reports of vascular complications in NF1 (77, 78).

### 3.7 Chemotherapy and radiotherapy

Chemotherapy is an option for unresectable or metastatic MPNST, with minimal response to adriamycin and ifosfamide (75). Doxorubicin is a first-line treatment, and its combination with ifosfamide shows the best response (79). Adjuvant chemotherapy has no impact on survival or recurrence (80), although epirubicin and ifosfamide may extend median survival from 45 to 75 months after local therapies (81). Radiation therapy is recommended for high-grade lesions or lesion larger than 5 cm (82) and provides excellent local control (83). Yet adjuvant radiation does not improve survival, despite some studies suggesting it can reduce tumor size for resection (84). Brachytherapy and intraoperative electron radiation therapy have also been utilized in MPNST therapy conventionally. Brachytherapy shows better local control than external beams and combining both may enhance results (85, 86).

## 4 Limitations

This report is inherently limited by its single-case nature and the short-term follow-up of twelve months, precluding definitive conclusions on long-term recurrence and survival. The highly specialized surgical approach described may not be generalizable to all centers. Future studies with larger cohorts and extended follow-up are needed to refine management guidelines. Nevertheless, this case underscores the critical importance of lifelong surveillance in NF1 patients and highlights the potential of integrated advanced techniques in managing these formidable tumors.

## 5 Conclusion

In summary, this case demonstrates the successful management of a rare, giant cervicothoracic MPNST in an NF1 patient through a meticulously planned, aggressive surgical strategy. It reinforces the necessity of a proactive, multidisciplinary approach for large, symptomatic MPNSTs, advocating for *en bloc* resection with vascular reconstruction to optimize oncological control and mitigate surgical risks. The strategic application of preoperative 3D modeling and selective embolization proved invaluable for planning and execution.

## Data Availability

The original contributions presented in the study are included in the article/Supplementary Material, further inquiries can be directed to the corresponding author.
